# The association between socioeconomic position, use of revascularization procedures and five-year survival after recovery from acute myocardial infarction

**DOI:** 10.1186/1471-2458-8-44

**Published:** 2008-02-01

**Authors:** Maria Rosvall, Basile Chaix, John Lynch, Martin Lindström, Juan Merlo

**Affiliations:** 1 Social Epidemiology, Department of Clinical Sciences, Malmö University Hospital, Lund University, Malmö, Sweden; 2 Inserm, U707, Paris, France; 3 Department of Epidemiology, Biostatistics and Occupational Health, McGill University, Montreal, Canada

## Abstract

**Background:**

Patients living under better socioeconomic circumstances often receive more active treatments after an acute myocardial infarction (AMI) compared to less affluent patients. However, most previous studies were performed in countries with less comprehensive coverage for medical services. In this Swedish nation-wide longitudinal study we wanted to evaluate long-term survival after AMI in relation to socioeconomic position (SEP) and use of revascularization.

**Methods:**

From the Swedish Myocardial Infarction Register we identified all 45 to 84-year-old patients (16,041 women and 30,366 men) alive 28 days after their first AMI during the period 1993 to 1996. We obtained detailed information on the use of revascularization, cumulative household income from the 1975 and 1990 censuses and 5-year survival after the AMI.

**Results:**

Patients with the highest cumulative income (adding the values of the quartile categories of income in 1975 and 1990) underwent a revascularization procedure within one month after their first AMI two to three times as often as patients with the lowest cumulative income and had half the risk of death within five years. The socioeconomic differences in the use of revascularization procedures could not be explained by differences in co-morbidity or type of hospital at first admission. Patients who underwent revascularization showed a similar lowered mortality risk in the different income groups, while there were strong socioeconomic differences in long-term mortality among patients who did not undergo revascularization.

**Conclusion:**

This nationwide Swedish study showed that patients with high income had a better long-term survival after recovery from their AMI compared to patients with low income. Furthermore, even though the use of revascularization procedures is beneficial, low SEP groups receive it less often than high SEP groups.

## Background

Low income has been associated with poorer survival after AMI [1-3]. Even in Sweden, despite a well-developed social welfare system, studies have shown socioeconomic differences in cardiac mortality rates [4]. Several potential explanations have been suggested, including, worse overall fitness [5], less compliance with drugs for secondary prevention [6], and less application of specialized cardiac state-of-the-art treatment among lower income groups [7,8].

Today, a growing number of patients recover after suffering their first-ever AMI [4,9] and it is relevant to understand which factors are related to increased long-term survival and to social differences in survival. During the last 20 years the role of risk factors (i.e., smoking, cholesterol and hypertension) in explaining the increase in survival after an AMI has decreased [10,11]. This is more pronounced in individuals in high socioeconomic position (SEP) and has been suggested to be partly attributed to greater benefits from treatment such as thrombolysis and revascularization procedures in this group [10,11]. The disability associated with heart failure after an AMI is largely a consequence of infarct size, and lack of timely treatment is a major determinant of increased infarct size [12]. Procedures such as invasive cardiac revascularization, including percutaneous transluminal coronary angioplasty (PTCA) and coronary artery bypass graft surgery (CABG), have been shown to increase survival [13-15] and there is some epidemiological evidence indicating that socioeconomic differences in survival after AMI is affected by differences in access and quality of hospital care [16].

In Sweden, all symptomatic AMI events are treated in public hospitals, where anybody can obtain treatment for a nominal fee (about 10 Euros per 24-hour period at the hospital covering medications, examinations and treatments during in-hospital care). After discharge the patient has to pay the first 185 Euros of the costs for medications for one year, where the government covers the costs exceeding this amount. In the present study, we examined data on all patients hospitalized with AMI in Sweden aged 45–84 years during the period 1993 to 1996. First, these data were used to determine whether socioeconomic position (as indicated by household income level) affected access to invasive cardiac procedures. Second, we examined the associations between cumulative income and mortality over five years after recovery from AMI. Even though it is known that patients living under poorer socioeconomic circumstances have reduced survival, the effect of socioeconomic conditions long before the AMI episode on survival is less known. Third, we examined the degree to which the use of revascularization affected socioeconomic differences in five-year mortality. Earlier studies from the United States, Great Britain, Canada and Finland have shown territorial inequalities according to which high income groups access more easily to large well-equipped, university hospitals [5,7,8,16-21]. However, few studies have specifically tried to investigate whether socioeconomic differences in the use of revascularization procedures explain or modulate differences in long-term mortality after an AMI [5,7,16]. Most of previous studies used aggregated data on SEP and none was nationwide. Furthermore, differences in the quality of hospital care might be related to the health insurance system of a country and might therefore not exist in countries with a universal health insurance system with comprehensive coverage for most medical and hospital services such as Sweden. All analyses were stratified by sex, since earlier Swedish studies have shown sex differences in incidence of AMI, case-fatality after an AMI and in the use of revascularization procedures, respectively [4,22].

## Methods

### Study population and information sources

During the period 1993 to 1996 a total of 60,680 patients aged 45 to 84 suffering their first AMI were admitted to acute care Swedish hospitals. Myocardial infarction was defined according to the International Classification of Diseases (ICD) 9th edition code 410. None of the patients were previously hospitalized for myocardial infarction (code 410), other acute and sub-acute forms of ischemic heart disease (code 411), or old myocardial infarction (code 412) – at least since 1987, from which time complete records of hospital diagnoses are available in Sweden. From this population we selected, 55,770 out of the 60,680 (92%) of the patients with complete information on income in 1990. Since the aim of our analysis was to investigate long-term survival in patients after the acute period following their first AMI, we further excluded 17% (9,363/55,770) of the patients dying within the first 28 days after hospital admission. Thus, the analyses were performed on 46,407 patients (16,041 women and 30,366 men).

For every patient we obtained information on discharge diagnoses from the National Myocardial Infarction Register [23] and on causes of death from the National Mortality Register at the Centre for Epidemiology (Swedish national Board of Health and Welfare) [24]. Coronary revascularization procedures (CABG or PTCA) were identified with use of procedure codes in the National Inpatient Register. Rates of revascularization procedures were examined for up to one month after the AMI, in order to allow for appropriate stratification of risk after the AMI and for waiting times. Coronary revascularization was defined as discharge with any operation code of '3066', '3067','3105', '3127', '3158', '3080', '3092' 'FNA', 'FNB', 'FNC', 'FND', 'FNE', 'FNF' or 'FNG'. Statistics Sweden [25] provided information on household income for the years 1975 and 1990, according to the Swedish censuses. The information on income in the Swedish censuses was register-based and collected from the Swedish register of individual income and wealth at Statistics Sweden. In order to preserve the anonymity of the subjects, the original personal identification number was encrypted before the database was provided to us by the relevant government authorities. This project was approved by the Data Safety Committees at Statistics Swedish Centre for Epidemiology, and by the Regional Ethical Review Board in Lund.

### Assessment of variables

#### Income variables

We used the information obtained from the 1975 and 1990 Swedish censuses on pre-tax household income during one year as an indicator of SEP. We calculated the equivalent household income by dividing household income by the number of people in the household, giving half the weight to children aged 17 and younger. Equivalent household income was classified into four groups by quartiles of household income: low (1), (for the 1975 census, i.e., 0 to 26 500 Swedish crowns or 0 to 4100 US dollars and for the 1990 census, i.e., 0 to 129 300 Swedish crowns or 0 to 19 900 US dollars); medium to low (2), (for the 1975 census, i.e., 26 500 to 43 200 Swedish crowns or 4100 to 6650 US dollars and for the 1990 census, i.e., 129 300 to 191 100 Swedish crowns or 19 900 to 29 400 US dollars); medium to high (3), (for the 1975 census, i.e., 43 200 to 58 000 Swedish crowns or 6650 to 8900 US dollars and for the 1990 census, i.e., 191 100 to 282 600 Swedish crowns or 29 400 to 43 500 US dollars); and high (4), (for the 1975 census, i.e., more than 58 000 Swedish crowns or 8900 US dollars and for the 1990 census, i.e., more than 282 600 Swedish crowns or 43 500 US dollars). By adding the values of the quartile categories of income in 1975 and 1990 for each patient, we created a cumulative income variable. This variable had a minimum value of two if the patient belonged to the low income group in both 1975 and 1990 (i.e., value = 1 + 1), and the maximum value of eight if the patient belonged to the high income group.

#### Previous hospitalisations

Previous hospitalisations were categorised in different diagnosis groups (ICD-9 codes within brackets) as cancer (140–239), diabetes (250), hypertension (401–405), angina pectoris and other forms of ischemic heart disease (413–414), cerebrovascular disease (430–438), and diseases of the respiratory system (460–519).

#### Type of hospital

Previous studies have demonstrated that admitting hospital characteristics are important determinants of the quality of care received [7,16]. Type of hospital at first hospital admission was therefore categorised into 'University hospital' and 'No university hospital'.

### Statistical methods

All patients were followed for a five-year period from 28 days after admission to the hospital between 1993 and 1996, until the follow-up ended five years later, in the period from 1998 to 2001 (or until the date of death, if it occurred before the five-year period ended).

Associations between cumulative income on one hand and previous hospitalisations, type of hospital at first hospital admission, hospital volume, and the use of revascularization procedures on the other hand, were investigated through age-adjusted logistic regression models stratified by sex.

We expressed the association between income groups and mortality by crude absolute risks (i.e., incidence density rates of deaths per 1000 person years), and by adjusted hazard ratios (95% CI) obtained from Cox Regression analysis [26]. We used robust standard error estimates in order to account for a possible hierarchical structure of the data [27]. However, since the hospital clustering was close to zero, this technique produced results similar to those obtained by conventional analytic methods [28]. In Model A we investigated the independent effect of income in 1975 and 1990 on five-year mortality risk by including both income variables in the same model. In Model B, we studied the effect of cumulative income. We also repeated the analysis by adjusting for previous hospitalisations. Parameters were estimated using SPSS version 11.5, STATA version 8, and MLwiN version 2.00 software packages [29].

Analyses of the associations between cumulative income and 5-year mortality after AMI were conducted separately among those who received and those who did not receive a revascularization in their first month after AMI. These analyses were adjusted for age, sex and previous hospitalisations and were performed separately for all ages, i.e., 45–84 years, those aged 45–64 years (65 years is the official retirement age in Sweden) and those aged 65 years or more.

## Results

### Study population

Table 1 shows that of the 46,407 first-time AMI patients who survived the first 28 days after admission to the hospital, 28% (8,577/30,366) of the men and 32% (5,194/16,041) of the women died during the ensuing five years. In total, 1014 (3.3%) men and 357 (2.2%) women received a coronary revascularization within one month after their first AMI. Table 2 and 3 shows age-adjusted previous hospitalizations, characteristics of hospital at first hospital admission and use of revascularization according to cumulative income in men and women, respectively. Low income groups generally had more previous hospitalizations, were less often admitted to a university hospital, and less often received revascularization than high income groups.

**Table 1:** Characteristics of a patient population aged 45 to 84 years admitted to the Swedish hospitals from 1993 to 1996 for first-time acute myocardial infarction (AMI), who survived the first 28 days after admission.

	Men	Women
Number of patients	30,366	16,041
Number of deaths during 5-year follow-up after surviving 28 days	8,577	5,194
Number of person years	124,888	64,149
Deaths per 1,000 person years (95% confidence interval)	69 (67–70)	81 (79–83)
Age in years (median, 1st to 3rd quartiles)	69 (61–76)	74 (67–79)
Revascularization within 1 month after the AMI (n; %)	1,014 (3.3)	357 (2.2)
Percentage of patients with previous hospitalizations (ICD codes):		
Cancer (140–239)	7	9
Diabetes (250)	6	10
Hypertension (401–405)	7	10
Angina pectoris and other ischemic heart disease (413–414)	15	17
Cerebrovascular disease (430–438)	7	8
Diseases of the respiratory system (460–519)	9	10

**Table 2:** Age-adjusted baseline characteristics of male patients aged 45 to 84 years that were hospitalized for first-time acute myocardial infarction in Swedish acute care facilities from 1993 to 1996 and who survived the first 28 days after admission (n = 30,366) according to cumulative income in the years 1975 and 1990.

	Cumulative income*							
	2 (n = 1,472)	3 (n = 2,489)	4 (n = 3,928)	5 (n = 5,750)	6 (n = 6,135)	7 (n = 5,914)	8 (n = 4,678)	*p for trend*
Percentage of patients with previous hospitalizations (ICD codes):								
Cancer (140–239)	6.7	7.6	8.9	9.5	8.7	8.2	7.3	0.25
Diseases of the respiratory system (460–519)	10.7	10.2	10.0	9.5	8.7	7.6	7.4	< 0.001
Diabetes (250)	9.2	9.1	8.0	6.5	5.9	5.0	4.4	< 0.001
Hypertension (401–405)	6.4	7.1	7.4	7.0	6.8	6.7	6.7	0.38
Angina pectoris/other ischemic heart disease (413–414)	17.3	16.7	16.1	16.2	15.5	13.8	14.0	< 0.001
Cerebrovascular disease (430–438)	8.1	7.6	8.0	7.4	7.2	6.6	6.4	0.001
University hospital, (%)	11.1	11.5	11.9	12.4	13.8	16.9	20.1	< 0.001
Hospital volume								
0 – 500	15.8	13.4	12.3	12.1	11.4	11.3	8.7	< 0.001
500 – 1500	32.9	34.3	35.1	34.3	32.8	31.8	28.7	< 0.001
1500 – 2500	46.4	47.5	48.2	48.2	49.1	48.5	52.9	< 0.001
> 2500	6.3	5.7	6.0	6.5	7.9	9.9	11.4	< 0.001
Revascularization† (%)	1.3	1.8	2.3	2.8	2.9	2.9	3.4	< 0.001

* By adding the values of the quartile categories of income in 1975 and 1990 for each patient, we created a cumulative income variable ranging from 2–8.

† Coronary revascularization was defined as discharge with any operation code '3066', '3067','3105', '3127', '3158', '3080', '3092' 'FNA', 'FNB', 'FNC', 'FND', 'FNE', 'FNF' or 'FNG' within one month after the first acute myocardial infarction.

**Table 3:** Age-adjusted baseline characteristics of female patients aged 45 to 84 years that were hospitalized for first-time acute myocardial infarction in Swedish acute care facilities from 1993 to 1996 and who survived the first 28 days after admission (n = 16,041) according to cumulative income in the years 1975 and 1990.

	Cumulative income*							
	2 (n = 4,346)	3 (n = 3,690)	4 (n = 2,770)	5 (n = 2,539)	6 (n = 1,364)	7 (n = 867)	8 (n = 465)	*p for trend*
Percentage of patients with previous hospitalizations (ICD codes):								
Cancer (140–239)	8.0	8.8	8.1	8.2	7.8	8.8	8.4	0.96
Diseases of the respiratory system (460–519)	10.0	9.5	9.1	8.5	7.8	8.7	6.1	0.001
Diabetes (250)	12.3	10.1	9.8	8.1	7.9	6.8	5.7	< 0.001
Hypertension (401–405)	10.4	9.5	9.8	7.9	8.0	8.6	7.2	0.001
Angina pectoris/other ischemic heart disease (413–414)	15.3	15.2	14.9	14.8	12.2	13.8	9.0	< 0.001
Cerebrovascular disease (430–438)	6.9	6.1	6.9	7.1	6.8	4.3	5.7	0.12
University hospital, (%)	10.4	12.0	14.8	15.4	20.4	23.5	31.2	< 0.001
Hospital volume								
0 – 500	14.4	12.4	11.4	12.0	9.5	9.7	6.2	< 0.001
500 – 1500	34.1	31.5	31.4	29.5	28.3	27.3	29.5	< 0.001
1500 – 2500	47.0	47.5	47.6	48.2	47.9	48.5	51.7	0.064
> 2500	5.3	7.4	8.7	9.2	12.8	15.4	13.1	< 0.001
Revascularization† (%)	1.2	2.1	2.3	2.7	2.7	3.1	2.1	< 0.001

* By adding the values of the quartile categories of income in 1975 and 1990 for each patient, we created a cumulative income variable ranging from 2–8.

† Coronary revascularization was defined as discharge with any operation code '3066', '3067','3105', '3127', '3158', '3080', '3092' 'FNA', 'FNB', 'FNC', 'FND', 'FNE', 'FNF' or 'FNG' within one month after the first acute myocardial infarction.

### Revascularization

Table 4 shows that those who had had coronary revascularization after their AMI were younger, more often male, more often had angina pectoris and hypertension and less often stroke, than those who had had no coronary revascularization.

**Table 4:** Age- and sex-adjusted means and prevalences of sociodemographic variables and previous hospitalizations by the use of revascularization among patients admitted to Swedish hospitals for first-time acute myocardial infarction from 1993 to 1996, who survived the first 28 days after admission.

	Revascularization	No Revascularization
		
	(n = 1,371)	(n = 45,036)
Age, years	63.8*	69.5
Male (%)	68.3*	64.8
Cancer (%)	6.4	6.8
Angina (%)	23.6*	14.7
Hypertension (%)	9.3*	7.6
Diabetes (%)	6.7	7.4
Stroke (%)	5.2*	6.9
Respiratory disease (%)	7.4	9.0

* P-values are given for the difference in sociodemographic variables and previous hospitalizations between those with and those without a coronary revascularization; *p < 0.05.

**Figure 1:**
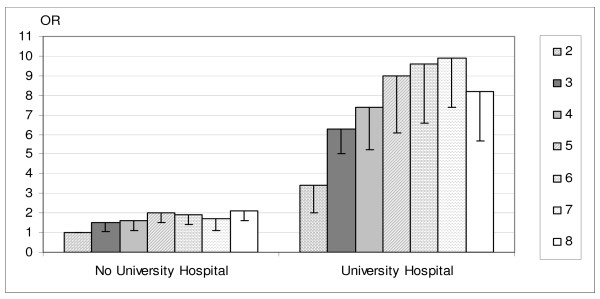
**Adjusted odds ratios (OR) of the use of revascularization procedures in all 30,366 men and 16,041 women aged 45 to 84 years that were hospitalized for their first acute myocardial infarction in Swedish acute care facilities from 1993 to 1996 and who survived the first 28 days after admission, by hospital at admission and cumulative income in 1975 and 1990.** Adjustments were made for age, sex and previous hospitalizations. Income was obtained by summing the values of the quartile categories of income in 1975 and in 1990. This variable has the minimum value of two if the patient belonged to the low income group (1) in both 1975 and 1990 (i.e., value = 1 + 1), and the maximum value of eight if the patient belonged to the high income group (i.e., value = 4 + 4). The lower borders of the 95% confidence intervals are marked.

Previous studies have demonstrated that admitting hospital characteristics are important determinants of the quality of care received [7,16]. Analyses of the associations between cumulative income and use of revascularization procedures, were therefore stratified by type of hospital. These analyses were adjusted for age, sex and previous hospitalisations (i.e., stroke, angina pectoris, respiratory disease, diabetes, hypertension and cancer). As seen in figure 1, the results showed a positive association between cumulative income and the use of revascularization both among those admitted to a university hospital and among those admitted to another type of hospital, with the largest relative differences among those admitted to a university hospital.

### Cumulative income and mortality

The crude association between income and mortality showed that the lower the income the higher the absolute risk of death (data not shown). The age-adjusted association between income and mortality is presented in table 5. Model A in table 5 show that in men mortality increased in a dose-response relation with decreasing income in both 1975 and in 1990, although the association was stronger for recent income. In women, however, mortality only increased significantly in relation to decreasing income in 1990. Model B in table 5 indicate that cumulative income was a good predictor of mortality in both men and women. We also repeated the analyses adjusting for previous hospitalisations, however, after this adjustment there was still an association between cumulative income and five year mortality among both men (p for trend < 0.001) and women (p for trend < 0.001). Similar results were seen when restricting the analyses to those aged 45–64 years and those aged 65 years or more, respectively.

**Table 5:** Age-adjusted hazard rate ratios (HRR) and 95 percent confidence intervals (95% CI) of mortality within five years after 28-days survival from first acute myocardial infarction in relation to income in 1975 and in 1990 as well as cumulative income 1975 and 1990 in 30,366 men and 16,041 women aged 45 to 84 years that were hospitalized in Swedish acute care facilities from 1993 to 1996.

	Men	Women
	HRR (95% CI)†	HRR (95% CI)†
*Model A*		
Independent income effects		
Income in 1990		
(4) high	1.00‡	1.00‡
(3) medium to high	1.23 (1.14, 1.32)	1.21 (1.06, 1.38)
(2) medium to low	1.43 (1.33, 1.54)	1.24 (1.09, 1.41)
(1) low	1.63 (1.51, 1.77)	1.44 (1.27, 1.63)
Income in 1975		
(4) high	1.00‡	1.00‡
(3) medium to high	1.02 (0.96, 1.08)	0.95 (0.83, 1.08)
(2) medium to low	1.10 (1.03, 1.17)	1.01 (0.91, 1.13)
(1) low	1.22 (1.13, 1.33)	1.07 (0.97, 1.18)
*Model B*		
Cumulative income 1975 and 1990*		
Sum 8	1.00‡	1.00‡
Sum 7	1.18 (1.08, 1.29)	1.97 (1.45, 2.68)
Sum 6	1.28 (1.17, 1.40)	1.66 (1.23, 2.23)
Sum 5	1.49 (1.37, 1.63)	1.81 (1.36, 2.41)
Sum 4	1.59 (1.46, 1.75)	1.86 (1.40, 2.47)
Sum 3	1.75 (1.59, 1.93)	2.00 (1.51, 2.65)
Sum 2	1.99 (1.79, 2.21)	2.24 (1.69, 2.97)

* Cumulative income is obtained by summing the values of the quartile categories of income in 1975 and in 1990. This variable has the minimum value of two if the patient belonged to the low income group (1) in both 1975 and 1990 (i.e., value = 1 + 1), and the maximum value of eight if the patient belonged to the high income group (i.e., value = 4 + 4).

† HRR, hazard rate ratio; CI, Confidence interval

‡ Reference category

Adjusting socioeconomic differences for the use of revascularization procedures only marginally reduced the age-adjusted HRRs of death within five years for the lowest cumulative income group compared to the highest, with in average 1% in men and women (data not shown). We also analyzed socioeconomic differences in survival through stratified analyses by use of revascularization procedures. This might be more appropriate since, in this population, only between 2–3% of those who recovered from their first AMI had a revascularization. We therefore divided the population into four groups, i.e., by cumulative income (divided into low and high at the median) and by the use of revascularization procedures. As seen in table 6, those with low cumulative income who had no revascularization showed an increased HRR of 5-year mortality, 1.35 (95% CI: 1.29, 1.40), compared to those with high cumulative income with no revascularization (reference group), after adjustment for age and sex. For those with high cumulative income who had a revascularization the HRR was 0.60 (95% CI: 0.48, 0.75). Having a low cumulative income and having had a revascularization was associated with an HRR of 0.69 (95% CI: 0.56, 0.86). Among those with revascularization there were no statistically significant mortality differences between those with high and low cumulative income. Adjustment for previous hospitalisations only marginally affected the results. Similar patterns of associations were seen among those aged 45–64 years and 65 years or more.

**Table 6:** Age- and sex-adjusted hazard rate ratios (HRR) with 95% confidence intervals for mortality within five years after 28-days survival from first acute myocardial infarction (AMI) by cumulative income and the use of revascularization procedures within the first month after the AMI in all 30,366 men and 16,041 women aged 45 to 84 years hospitalized in Swedish acute care facilities from 1993 to 1996.

	No revascularization§		Revascularization§	
		
	High income†‡ HRR (95% CI)*	Low income‡ HRR (95% CI)	High income‡ HRR (95% CI)	Low income‡ HRR (95% CI)
*All ages*				
Age and sex adjusted	(cases n = 3,886) 1.00‡¶	(cases n = 9,722) 1.35 (1.29, 1.40)¶	(cases n = 78) 0.60 (0.48, 0.75)	(cases n = 85) 0.69 (0.56, 0.86)
*45–64 years*				
Age and sex adjusted	(cases n = 638) 1.00‡	(cases n = 639) 1.75 (1.55, 1.96)¶	(cases n = 25) 0.73 (0.50, 1.10)	(cases n = 23) 1.39 (0.90, 2.10)
*65 years or more*				
Age and sex adjusted	(cases n = 3,248) 1.00‡¶	(cases n = 9,083) 1.29 (1.23, 1.34)¶	(cases n = 53) 0.57 (0.43, 0.74)	(cases n = 62) 0.58 (0.45, 0.75)

* HRR, Hazard rate ratio; CI, confidence interval.

† Reference category

‡ By adding the values of the quartile categories of income in 1975 and 1990 for each patient, we created a cumulative income variable ranging from 2–8. This measure was divided into high and low income at the median, i.e., the value of 5.

§Coronary revascularization was defined as discharge with any operation code of '3066', '3067','3105', '3127', '3158', '3080', '3092','FNA', 'FNB', 'FNC', 'FND', 'FNE', 'FNF' or 'FNG' within one month after the first myocardial infarction.

¶Statistcically significant (p < 0.05) compared to those with high income who had revascularization.

## Discussion

There is some epidemiological evidence indicating that socioeconomic differences in survival after an AMI is affected by differences in access and quality of hospital care [16]. This mechanism should be less relevant in Sweden, with equalitarian access to health care resources. Nevertheless, socioeconomic differences are still observable. In this national study, we found effects of SEP measured as household income on the rate of invasive cardiac procedures, as well as on mortality five years after recovery from the first AMI. 

Also studies from other countries have shown that low SEP groups receive less active treatments after an AMI than high SEP groups [5,7,8,16-21], even though there are some negative studies [1,30]. It is not likely that the socioeconomic differences seen in our study are explained by less severe disease among patients in low SEP groups, since one would rather expect these patients to have more severe coronary artery disease due to a higher prevalence of cardiovascular risk factors [4]. On the other hand, it might be that low income groups show a too deteriorated clinical picture to be able to undergo revascularisation. However, studies have shown that AMI patients admitted to invasive-procedure hospitals have a more adverse risk profile than patients admitted to noninvasive-procedure hospitals [31] and even after adjustment for previous hospitalizations, there was still an association between cumulative income and the use of revascularization procedures. One explanation might be that higher income groups more often live near university hospitals with more advanced treatment methods than local hospitals, and might therefore more easily receive timely effective treatment. But, in our study, the socioeconomic differences in the use of revascularization procedures was greater among those admitted to a university hospital than among those admitted to another type of hospital. Another plausible explanation might be that low income groups arrive too late at hospital to undergo a revascularisation. A recent review article by Moser et al. on different treatment-seeking delay phases for the acute coronary syndrome, showed that low SEP is associated with increased delays in seeking treatment [12]. 

Differences in the use of revascularization procedures could, however, not explain the socioeconomic differences in 5-year mortality. These results are in line with the findings from other studies. In a prospective study on 2,142 AMI patients from 18 hospitals in the United States, the relationship between household income and one-year mortality was only slightly attenuated after adjustment for quality of care including reperfusion [5]. In a Canadian study including all 51,591 patients with AMI admitted to hospitals in Ontario during 1994 to 1997, differences in access to coronary revascularization did not account for income-related (measured as neighborhood median household income) differences in 1-year mortality [7]. In a retrospective Canadian study, of 5,622 patients attending the hospital emergency department with an AMI between 1998 and 2002 in the Province of Alberta, those with low SEP (i.e., low neighbourhood household income) had an increased 1-year mortality, even after adjustment for baseline characteristics and 1-year revascularization [16].

The rate of invasive cardiac procedures was rather low in Sweden during the mid-1990s [32]. For example, in 1995 only between 3–5% of the AMI patients in the ages 65–74 years had a primary PCI after an AMI with an increased ST-segment or a left bundle blockage. Since then there has been a sharp increase in the use of primary PCI and the corresponding figures in 2006 were between 55–57%. Since, in our study (excluding patients dying within the first 28 days after hospital admission) on average only 3.3% of the men and 2.2% of the women having a first AMI had a revascularization within one month, we also performed stratified analyses by the use of revascularization procedures. Among those who had had no revascularization, low income groups had a higher mortality than high income groups. However, those who underwent revascularization generally had a lower mortality rate with no socioeconomic mortality differences. Similar results were seen in the retrospective study from Canada, where the impact of SEP on mortality was largely confined to non-revascularized patients [16] and in a Scottish study on 1,346 consecutive patients undergoing their first PTCA, 1-year mortality were similar for patients in different SEP groups [33]. This could be due to an effectiveness of revascularization irrespective of SEP. It could also be due to physicians selecting a homogeneous population of patients in terms of clinical characteristics, who are clinically appropriate for revascularisation. Furthermore, it could be that those having a revascularization are more likely to be followed up by a cardiologist than a general practitioner and more likely to receive specific secondary prevention advice and a prescription for aspirin, β-blockers, angiotensin-converting enzyme inhibitors, or statins following discharge [31]. This could be of a higher benefit to lower income groups with worse risk factor profiles.

Although more affluent areas tend to have a greater concentration of specialized services, inequitable distribution of hospital resources did not account for the effects of SEP on mortality after an AMI. These results are in line with results from other studies [34,35]. In Sweden there are observable differences among hospitals in the proportion of patients treated with intravenous beta-blockers, intravenous nitroglycerin, intravenous or subcutaneous anticoagulants, and lipid-lowering medication, and even larger discrepancies in echocardiography and revascularization within 14 days [36,37]. These hospital disparities seem associated with hospital differences in patient survival in some studies [36], but not in others [37]. In order to quantify hospital variance in five-year mortality, we applied multilevel logistic regression [27,38]. This variance appeared to be close to zero, which suggests that hospital practice variation has minimal relevance for understanding *long-term *mortality differences among AMI patients who survive four weeks after admission. However, other contexts like the small local areas where the individual live might condition survival over and above individual characteristics [39], through mechanisms related to for example practical support.

Regarding other causal pathways between SEP and survival after AMI, low income is an early determinant of major cardiovascular risk factors [40-42] as well as the development of other diseases such as diabetes, respiratory diseases, and other cardiovascular diseases, which in turn affect survival after an AMI. Since, mentioned above, the rate of invasive procedures was rather low in the mid-1990s, these factors have probably contributed in a higher degree to the higher mortality rates in low income groups. We repeated the analyses with adjustments for previous hospitalization concerning a large number of relevant diagnoses, and differences in co-morbidity measured in this way could not explain the results. However, there might still be differences in non-hospitalized co-morbidity. It could also be that individuals in lower SEP have larger infarctions and therefore a worse prognosis [43,44]. However, there are also negative studies [8]. Low income is also related to greater distances to hospitals, potentially leading to longer time delays before diagnosis and treatment. All these factors might, rather than being confounders, be along the pathway between low income and mortality.

### Strengths and limitations of the study

There are several strengths of our study. Firstly, our data covers the total general population in Sweden, which minimized the risk for selection bias. Therefore, the common problem of many previous studies based on a selected sample of healthy people with the potential attenuation of the associations, is not present in our study. The study population in our study was restricted to patients without any hospitalization for AMI for at least seven years prior to hospital admission and those patients with a coexisting diagnosis of old AMI were excluded. These restrictions increase the homogeneity of the population regarding underlying cardiovascular disease severity. Secondly, Sweden has a long tradition of administering health care registers, and their quality is regularly audited. Moreover, the existence of a centralised national registry limits the risk of differential information bias that might affect findings if locally-based (e.g., county) registers were used [45]. The validity of cases of AMI recorded by the Swedish Myocardial Infarction Register has been investigated and judged to be acceptable for epidemiological analysis [46]. The Myocardial Infarction Register has very wide coverage, comprising all persons with AMI included in the study period reported either to the Cause of Death Register (which covers all Swedish residents, whether or not their death occurred in Sweden), or to the Hospital Discharge Register (which includes all patients discharged from hospitals in Sweden). The mortality register encompass 97% of all deaths in Sweden, while census participation rates range between 97% and 99%. Therefore, an advantage of our analysis was the possibility of investigating all patients in Sweden using valid information on AMI diagnoses and detailed census data on income over a long period of time. Consequently, our analysis provides unique evidence on how socioeconomic characteristics during the last two decades shape survival after recovery for first-time AMI patients for many years to come.

However, there were limitations to our study. Firstly, we had no information on health-damaging behaviour, healthcare utilisation or social support, preventing us from investigating the mediating mechanisms of the income-mortality association and also potential confounders for the income-revascularization association. Secondly, admitting hospital characteristics were stratified into university hospital status or not. However, a more detailed categorization based on the revascularization capability of all hospitals would have been preferable. Third, we had no information on the income at the time when the AMI occured. However, changes in income level during the period 1990 to 1993–1996 would probably result in a non-differential misclassification leading to a reduction in the associations studied. Furthermore, the longitudinal design allowed us to use multiple predictors of income over the life course, which would be expected to be associated with less measurement error than using single measures. Fourth, since the study sample was designed to allow us to investigate long-term survival in patients who have passed through the critical four weeks after an AMI, we excluded those who died within 4 weeks after their AMI. Excluding these patients, could, however, lead to a weakening of an association between SEP and survival since earlier studies have shown an inverse relationship between SEP and short-term survival after an AMI [47].

### Future directions

In this nationwide study, we found that patients with high income had a better long-term survival after recovery from their AMI compared to patients with low income. Even though the mechanisms behind the association need to be further established, nurses and physicians should recognize low SEP as an indicator of worse prognosis after an AMI. Furthermore, although Sweden is a country with equalitarian access to health care resources, our study suggests that there are differences in the quality of hospital care measured as the rate of revascularization after a first AMI. The sources of such differential selection processes by SEP remain unclear, but their existence will probably influence the present debate about the future organization of the Swedish health care system.

## Conclusion

Despite Sweden's well-developed social welfare system, those with higher cumulative income during the decades before an AMI event underwent invasive procedures after the first AMI more than twice as often as lower-income groups. Patients who underwent revascularization showed a similar lowered mortality risk in different income groups, while there were strong socioeconomic differences in the long-term mortality among patients who did not undergo revascularization. Thus, even though the use of revascularization procedures is effective and seem to be equity enhancing, low SEP groups receive it less often than high SEP groups. The sources of such differential selection processes by SEP remain unclear, but their existence is one of many challenges to the equity goals of the Swedish welfare state.

## Competing interests

The author(s) declare that they have no competing interests.

## Authors' contributions

MR, BC and JM have contributed to the conception and design of the work, the acquisition of data, the analysis of the data, the interpretation and the discussion of the results. JL and ML have contributed to the conception and design of the work, the interpretation and the discussion of the results. All authors read and approved the final manuscript.

## Pre-publication history

The pre-publication history for this paper can be accessed here:



## References

[B1] Rao SV, Schulman KA, Curtis LH, Gersh BJ, Jollis JG (2004). Socioeconomic status and outcome following acute myocardial infarction in elderly patients. Arch Intern Med.

[B2] Alter DA, Chong A, Austin PC, Mustard C, Iron K, Williams JI, Morgan CD, Tu JV, Irvine J, Naylor CD, SESAMI Study Group (2006). Socioeconomic status and mortality after acute myocardial infarction. Ann Intern Med.

[B3] Davey Smith G, Lynch JW, Kuh D, Ben-shlomo (2004). Life course approaches to socioeconomic differentials in health. A Lifecourse Approach to Chronic Disease Epidemiology.

[B4] National Board of Health and Welfare (2005). Public Health Report of Sweden (in Swedish).

[B5] Bernheim SM, Spertus JA, Reid K, Bradley E, Desai R, Peterson E, Rathore S, Normand S-L, Jones P, Rahimi A, Krumholz H (2007). Socioeconomic disparities in outcomes after acute myocardial infarction. Am Heart J.

[B6] Ho PM, Spertus JA, Masoudi FA, Reid KJ, Peterson ED, Magid DJ, Krumholz HM, Rumsfeld JS (2006). Impact of medication therapy discontinuation on mortality after myocardial infarction. Arch Intern Med.

[B7] Alter DA, Naylor CD, Austin P, Tu JV (1999). Effects of socioeconomic status on access to invasive cardiac procedures and on mortality after acute myocardial infarction. N Engl J Med.

[B8] Salomaa V, Miettinen H, Niemelä M, Ketonen M, Mähönen M, Immonen-Räihä P, Lehto S, Vuorenmaa T, Koskinen S, Palamäki P, Mustaniemi H, Kaarsalo E, Arstila M, Torppa J, Kuulasmaa K, Puska P, Pyörälä K, Tuomilehto J (2001). Relation of socioeconomic position to the case fatality, prognosis and treatment of myocardial infarction events; the FINMONICA MI Register Study. J Epidemiol Community Health.

[B9] Abildstrom SZ, Rasmussen S, Rosen M, Madsen M (2003). Trends in incidence and case fatality rates of acute myocardial infarction in Denmark and Sweden. Heart.

[B10] Vartiainen E, Pekkanen J, Koskinen S, Jousilahti P, Salomaa V, Puska P (1998). Do changes in cardiovascular risk factors explain the increasing socioeconomic difference in mortality from ischemic heart disease in Finland. J Epidemiol Community Health.

[B11] Harald K, Pajunen P, Jousilahti P, Koskinen S, Vartiainen E, Salomaa V (2006). Modifiable risk factors have an impact on socio-economic differences in coronary heart disease events. Scand Cardiovasc J.

[B12] Moser D, Kimble L, Alberts M, Alonzo A, Croft J, Dracup K, Evenson K, Go A, Hand M, Kothari R, Mensah G, Morris D, Pancioli A, Riegel B, Zerwic J (2006). Reducing Delay in Seeking Treatment by Patients With Acute Coronary Syndrome and Stroke. A Scientific Statement From the American Heart Association Council on Cardiovascular Nursing and Stroke Council. Circulation.

[B13] Grines C, Patel A, Zijlstra F, Weaver WD, Granger C, Simes RJ, PCAT Collaborators (2003). Percutaneous transluminal coronary angioplasty. Primary coronary angioplasty compared with intravenous thrombolytic therapy for acute myocardial infarction: six-month follow up and analysis of individual patient data from randomized trials. Am Heart J.

[B14] Zijlstra F, Hoorntje JC, de Boer MJ, Reiffers S, Miedema K, Ottervanger JP, van 't Hof AW, Suryapranata H (1999). Long-term benefit of primary angioplasty as compared with thrombolytic therapy for acute myocardial infarction. New Engl J Med.

[B15] (1999). Invasive compared with non-invasive treatment in unstable coronary-artery disease. FRagmin and Fast Revascularisation during InStability in Coronary artery disease Investigators. Lancet.

[B16] Chang W-C, Kaul P, Westerhout C, Graham M, Armstrong P (2007). Effects of socioeconomic status on mortality after acute myocardial Infarction. Am J Med.

[B17] Miettinen H, Salomaa V, Ketonen M, Niemelä M, Immonen-Räihä P, Mähönen M (1999). Trends in the treatment of patients with myocardial infarction and coronary revascularization procedures in Finland during 1986–92: the FINMONICA Myocardial Infarction Register Study. J Intern Med.

[B18] Philbin EF, McCullough PA, DiSalvo TG, Dec GW, Jenkins PL, Weaver WD (2000). Socioeconomic status is an important determinant of the use of invasive procedures after acute myocardial infarction in New York State. Circulation.

[B19] Hetemaa T, Keskimäki I, Salomaa V, Mahonen M, Manderbacka K, Koskinen S (2004). Socioeconomic inequities in invasive cardiac procedures after first myocardial infarction in Finland in 1995. J Clin Epidemiol.

[B20] Alter DA, Iron K, Austin PC, Naylor CD, SESAMI Study Group (2004). Socioeconomic status, service patterns, and perceptions of care among survivors of acute myocardial infarction in Canada. JAMA.

[B21] Morris RW, Whincup PH, Papacosta O, Walker M, Thomson A (2005). Inequalities in coronary revascularisation during the 1990s: evidence from the British regional heart study. Heart.

[B22] (2004). Jämställd vård? Könsperspektiv på hälso-och sjukvården: In Swedish.

[B23] Centre for Epidemiology, National Board of Health and Welfare (1998). Myocardial infarctions in Sweden 1987–1996 Artikelnr 1998-42-006.

[B24] National Mortality Register The National Board of Health and Welfare. Centre for Epidemiology. http://www.socialstyrelsen.se/statistik/statistik_amne/dodsorsaker/index.htm.

[B25] Statistics Sweden. http://www.scb.se/.

[B26] Yang M, Goldstein H (2003). Modelling survival data in MLwiN 1.20. MLwiN 120 users manual Centre for Multilevel Modelling, Bedford Group for Lifecourse and Statistical Studies, Institute of Education.

[B27] Austin PC, Tu JV, Alter DA (2003). Comparing hierarchical modeling with traditional logistic regression analysis among patients hospitalized with acute myocardial infarction: should we be analyzing cardiovascular outcomes data differently?. Am Heart J.

[B28] Goldstein H (2003). Multilevel Statistical Models.

[B29] Browne WJ (2003). MCMC Estimation in MLwiN Version 20.

[B30] Britton A, Shipley M, Marmot M, Hemingway H (2004). Does access to cardiac investigation and treatment contribute to social and ethnic differences in coronary heart disease? Whitehall II prospective cohort study. BMJ.

[B31] Alter D, Naylor CD, Austin P, Tu J (2001). Long-term MI outcomes at hospitals with or without on-site revascularization. JAMA.

[B32] Stenestrand U, Wallentin L (2007). RIKS-HIA & SEPHIA ÅRSRAPPORT 2006 (RIKS-HIA & SEPHIA REPORT OF 2006 (In Swedish), Uppsala. http://www.ucr.uu.se/rikshia.

[B33] Denvir MA, Lee AJ, Rysdale J, Walker A, Eteiba H, Starkey IR, Pell JP (2006). Influence of socioeconomic status on clinical outcomes and quality of life after percutaneous coronary intervention. J Epidemiol Community Health.

[B34] Every NR, Parsons LS, Fihn SD, Larson EB, Maynard C, Hallstrom AP, Martin JS, Weaver WD (1997). Long-term outcome in acute myocardial infarction patients admitted to hospitals with and without on-site cardiac catheterization facilities. Circulation.

[B35] Pilote L, Califf RM, Sapp S, Miller DP, Mark DB, Weaver WD, Gore JM, Armstrong PW, Ohman EM, Topol EJ (1995). Regional variation across the United States in the management of acute myocardial infarction. GUSTO-1 Investigators. Global Utilization of Streptokinase and Tissue Plasminogen Activator for Occluded Coronary Arteries. N Engl J Med.

[B36] Stenestrand U, Lindback J, Wallentin L (2005). Hospital therapy traditions influence long-term survival in patients with acute myocardial infarction. Am Heart J.

[B37] Wallentin L, Spångberg K, Lindbäck J, Hagerman I, Hansen O, Held C, Karlson JE, Matsson E, Mooe T, Näslund U, Sterner M, Svenberg L, Werner U, Stenestrand U for the RIKS-HIA group in Sweden (2004). UCR-rapport. Öppen redovisning av vårdinsatser och resultat vid behandling av akut hjärtinfarkt på olika sjukhus i Sverige 2003 [Open presentation of the results from health care interventions and treatment of acute myocardial infarction in different hospitals in Sweden 2003].

[B38] Merlo J, Ostergren P-O, Broms K, Bjorck-Linne A, Liedholm H (2001). Survival after initial hospitalisation for heart failure: a multilevel analysis of patients in Swedish acute care hospitals. J Epidemiol Community Health.

[B39] Chaix B, Rosvall M, Merlo J (2007). Neighborhood socioeconomic deprivation and residential instability: effects on incidence of ischemic heart disease and survival after AMI. Epidemiology.

[B40] Alter DA, Iron K, Austin PC, Naylor CD, SESAMI Study Group (2004). Influence of education and income on atherogenic risk factor profiles among patients hospitalized with acute myocardial infarction. Can J Cardiol.

[B41] Kaplan GA, Keil JE (1993). Socioeconomic factors and cardiovascular disease: a review of the literature. Circulation.

[B42] Dallongeville J, Cottel D, Ferrieres J, Arveiler D, Bingham A, Ruidavets JB, Haas B, Ducimetiere P, Amouyel P (2005). Household income is associated with the risk of metabolic syndrome in a sex-specific manner. Diabetes Care.

[B43] Tofler GH, Muller JE, Stone PH, Davies G, Davis VG, Braunwald E (1993). Comparison of long-term outcome after acute myocardial infarction in patients never graduated from high school with that in more educated patients. Multicenter Investigation of the Limitation of Infarct Size (MILIS). Am J Cardiol.

[B44] Barakat K, Stevenson S, Wilkinson P, Suliman A, Ranjadayalan K, Timmis AD (2001). Socioeconomic differentials in recurrent ischaemia and mortality after acute myocardial infarction. Heart.

[B45] Merlo J, Lindblad U, Pessah-Rasmussen H, Hedblad B, Rastam J, Isacsson SO, Janzon L, Rastam L (2000). Comparison of different procedures to identify probable cases of myocardial infarction and stroke in two Swedish prospective cohort studies using local and national routine registers. Eur J Epidemiol.

[B46] (2000). Värdering av diagnoskvaliteten för akut hjärtinfarkt i patientregistret 1987 och 1995 [Validation of the acute myocardial infarction diagnosis quality in the hospital patient register 1987–1995].

[B47] Morrison C, Woodward M, Leslie W, Tunstall-Pedoe H (1997). Effect of socioeconomic group on incidence of, management of, and survival after myocardial infarction and coronary death: analysis of community coronary event register. BMJ.

